# IQGAP Family Proteins in Colorectal Cancer: Molecular Mechanisms and Prognostic Implications

**DOI:** 10.3390/diagnostics16142153

**Published:** 2026-07-09

**Authors:** Catherine Keiko Gunawan, Anton Sumarpo, Budiono Raharjo, Ida Parwati

**Affiliations:** 1 Medical Profession Study Program, Faculty of Medicine, Maranatha Christian University, Bandung 40164, Indonesia; c.keiko.g@gmail.com; 2 Department of Clinical Pathology, Faculty of Medicine, Maranatha Christian University, Bandung 40164, Indonesia; 3 Department of Clinical Pathology, Faculty of Medicine, Universitas Wijaya Kusuma Surabaya, Surabaya 60225, Indonesia; dr.budi.sby81@gmail.com; 4 Department of Clinical Pathology, Faculty of Medicine, Universitas Padjadjaran—Hasan Sadikin Hospital, Bandung 40161, Indonesia; ida.parwati@unpad.ac.id

**Keywords:** colorectal cancer, IQGAP1, IQGAP2, IQGAP3

## Abstract

Colorectal cancer (CRC) remains a leading cause of cancer-related morbidity and mortality worldwide, and the molecular mechanisms driving its progression and metastasis remain incompletely understood. Increasing evidence highlights the role of IQ motif-containing GTPase-activating proteins (IQGAPs)—a family of scaffold proteins consisting of IQGAP1, IQGAP2, and IQGAP3—in regulating multiple signaling pathways involved in tumor development. This narrative review provides an overview of the molecular roles of IQGAP family proteins in CRC, focusing on their involvement in oncogenic signaling and their potential prognostic significance. Current evidence suggests that IQGAP family members play distinct and sometimes opposing roles in CRC biology. IQGAP1 is frequently overexpressed and acts as a scaffold that activates ERK/MAPK and β-catenin signaling, promoting proliferation, invasion, and metastasis. In contrast, IQGAP2 exhibits tumor-suppressive properties, with reduced expression associated with more aggressive disease and unfavorable outcomes. Meanwhile, IQGAP3 has emerged as a potential oncogenic driver, where its upregulation correlates with enhanced tumor growth, metastatic potential, and poorer survival, possibly through PI3K-related signaling. Together, these findings highlight the context-dependent roles of IQGAP proteins in CRC and suggest their potential as novel biomarkers and therapeutic targets. Further validations are needed to explore the clinical utility of IQGAP proteins in CRC.

## 1. Introduction

Colorectal cancer (CRC) is the third most commonly diagnosed malignancy and the second leading cause of cancer-related mortality worldwide, accounting for approximately 9.4% of all cancer deaths [1,2]. Despite advances in early detection and treatment, the global incidence continues to rise, with projections estimating 3.2 million new cases by 2040, driven by aging populations and changing lifestyle risk factors in developing regions [3]. A major contributor to CRC mortality is metastatic progression, which arises from a complex interplay of molecular, genetic, and epigenetic alterations, together with the biological heterogeneity of the disease [3,4,5]. Given this rising epidemiological burden, a deeper understanding of the molecular mechanisms underlying CRC progression is urgently needed [6].

CRC develops through a multistep process in which the gradual accumulation of genetic and epigenetic alterations transforms normal colonic epithelium into benign polyps, adenomas, and ultimately invasive carcinomas [7,8]. These alterations dysregulate key signaling pathways that not only initiate tumorigenesis but also sustain aberrant proliferation, survival, and metastatic spread [9,10,11]. Among the most prominent pathways implicated in CRC pathogenesis are the Wnt/β-catenin, RAS/RAF/MEK/ERK, phosphoinositide 3-kinase (PI3K)/AKT, and transforming growth factor-β (TGF-β) cascades, which collectively regulate differentiation, apoptosis, migration, and angiogenesis, and whose dysregulation is frequently associated with tumor progression [6,12,13,14,15,16].

IQ motif-containing GTPase-activating proteins (IQGAPs), comprising three related isoforms—IQGAP1, IQGAP2, and IQGAP3—have emerged as important scaffold proteins that integrate multiple cellular signaling networks [17,18]. Through interactions with components of the Wnt/β-catenin, ERK/MAPK, and PI3K/AKT pathways, IQGAPs regulate essential cellular processes including proliferation, migration, adhesion, cytoskeletal dynamics, and epigenetic regulation [18,19,20]. However, despite growing interest and a substantial body of research, the precise mechanisms through which IQGAP proteins contribute to CRC progression and clinical outcomes remain incompletely understood [21].

This narrative review summarizes current evidence on the molecular roles of IQGAP family proteins in colorectal cancer, with particular emphasis on their involvement in oncogenic signaling pathways and their potential clinical implications. We highlight the distinct and sometimes opposing roles of IQGAP1, IQGAP2, and IQGAP3 in CRC biology, and discuss their emerging potential as prognostic biomarkers and therapeutic targets.

## 2. Biological Functions and Structural Features of IQGAP Proteins

### 2.1. Biological Functions and Molecular Characteristics of IQGAP Proteins

IQ motif-containing GTPase-activating proteins (IQGAPs) constitute an evolutionarily conserved family of scaffold proteins that interact with a wide range of molecular partners to regulate diverse cellular processes [22]. These processes include cytokinesis, cell migration, proliferation, intracellular signaling, vesicle trafficking, and cytoskeletal organization. Through their ability to integrate multiple signaling pathways, IQGAP proteins play a crucial role in coordinating cellular responses to environmental and intracellular cues [17].

IQGAP proteins are widely distributed across eukaryotic organisms, ranging from fungi and protists to higher animals. In vertebrates, including humans, three closely related isoforms have been characterized: IQGAP1, IQGAP2, and IQGAP3 [23]. Although these proteins share considerable structural similarity, accumulating evidence suggests that they exhibit distinct biological functions and regulatory roles depending on cellular context and tissue type [24,25,26].

Among the three isoforms, IQGAP1 is the most extensively studied and has been implicated in the regulation of multiple signaling pathways associated with cell migration, cytoskeletal remodeling, and tumor progression [27]. In contrast, IQGAP2 has been reported to exhibit tumor-suppressive properties in several malignancies, whereas IQGAP3 is increasingly recognized for its involvement in promoting cell proliferation and tumor aggressiveness [28,29,30]. The functional diversity of these proteins highlights the complex regulatory roles of the IQGAP family in maintaining cellular homeostasis, as well as their potential contribution to cancer development. Given their ability to function as molecular scaffolds that coordinate multiple signaling networks, increasing attention has been directed toward understanding the involvement of IQGAP family proteins in cancer biology, including CRC progression and prognosis.

### 2.2. Structural Domains of IQGAP Proteins

A defining feature of IQGAP proteins is their conserved multidomain architecture, which enables them to function as scaffold molecules coordinating diverse signaling and cytoskeletal processes [18]. Despite exhibiting sequence similarities to Ras GTPase-activating proteins (RasGAPs), IQGAP proteins lack intrinsic GAP catalytic activity. Instead, their structural organization allows them to facilitate interactions among cytoskeletal components, small GTPases, and intracellular signaling molecules, thereby integrating multiple cellular signaling pathways [31].

In mammals, IQGAP proteins typically contain several conserved structural domains, including the N-terminal calponin homology domain (CHD), the WW domain, multiple IQ motifs, the Ras GTPase-related domain (GRD), and the RasGAP C-terminal (RGCT) domain [22]. The CHD mediates direct binding to filamentous actin (F-actin), contributing to cytoskeletal organization and dynamic cellular processes such as migration and adhesion [32,33]. Adjacent to this region are IQGAP-specific repeats (Irs), whose precise functional roles remain incompletely understood but are believed to contribute to structural stability and regulatory interactions within the protein complex [34].

The WW domain serves as a protein–protein interaction module capable of recognizing proline-rich motifs in target proteins [35]. Structurally, WW domains adopt a compact three-stranded β-sheet conformation and typically bind motifs such as PPXY, PPPR, or phosphorylated Ser/Thr-Pro sequences [36]. Although earlier studies proposed that ERK1/2 interact with the WW domain of IQGAP1, subsequent work has challenged this model, indicating that the WW domain is neither necessary nor sufficient for this interaction [37,38].

IQGAP proteins also contain multiple IQ motifs, which function as binding sites for calmodulin and other calcium-dependent regulatory proteins. Through these interactions, IQ motifs allow IQGAP proteins to participate in calcium-mediated signaling processes that influence cytoskeletal dynamics and cellular responses to intracellular signals [26].

Another critical structural element is the Ras GTPase-related domain (GRD). Although this domain exhibits strong structural homology to RasGAP domains found in canonical RasGAP proteins, IQGAP proteins lack GAP enzymatic activity due to mutations in key catalytic residues. Instead, the GRD, together with the RGCT domain, mediates interactions with small Rho family GTPases such as Rac1 and Cdc42, which are essential regulators of cytoskeletal organization, cell polarity, and migration [39,40,41].

The RGCT domain, located at the C-terminus, further contributes to the functional versatility of IQGAP proteins by interacting with several signaling and adhesion molecules, including E-cadherin, β-catenin, and phosphatidylinositol 4,5-bisphosphate (PIP2) [42]. In addition, this region contains phosphorylation sites that regulate IQGAP activity and modulate its ability to assemble signaling complexes [34]. Collectively, the coordinated functions of these structural domains enable IQGAP proteins to serve as central scaffolds that spatially organize signaling networks linking cytoskeletal regulation with intracellular signaling pathways.

### 2.3. IQGAP Proteins as Signaling Scaffolds

The multidomain structure of IQGAP proteins enables them to mediate protein–protein interactions with a wide range of binding partners involved in diverse signaling pathways. As scaffold proteins, IQGAPs facilitate the assembly of signaling complexes by bringing multiple pathway components into proximity, thereby enhancing the efficiency and specificity of intracellular signal transduction [17]. Although scaffold proteins are not direct mediators of enzymatic signaling reactions, they play a crucial role in organizing signaling molecules within the highly crowded and heterogeneous cytosolic environment, allowing biochemical processes to occur in a spatially and temporally coordinated manner.

Scaffold proteins are widely involved in the regulation of multiple intracellular signaling pathways, including the mitogen-activated protein kinase (MAPK) and Wnt signaling cascades [43,44,45,46,47]. Similar to other scaffold proteins, IQGAP family members function as central signaling hubs that integrate interactions between cytoskeletal components, small GTPases, and signaling molecules [26]. Through these interactions, IQGAP proteins have been implicated in the regulation of key pathways such as ERK/MAPK, phosphoinositide 3-kinase (PI3K)/AKT, Wnt/β-catenin signaling, and Rho family GTPase-mediated processes involving Rac1 and Cdc42. These pathways are critically involved in the regulation of cellular processes, including proliferation, migration, adhesion, and cytoskeletal remodeling [48].

By coordinating multiple signaling networks, IQGAP proteins contribute to the maintenance of cellular homeostasis and to the regulation of cellular responses to extracellular stimuli [22,49]. Given the central role of these signaling pathways in tumor development and progression, increasing attention has been directed toward understanding the involvement of IQGAP-mediated signaling networks in cancer biology, including CRC progression.

## 3. IQGAP Family in Colorectal Cancer Progression and Prognosis

**Figure 1:**
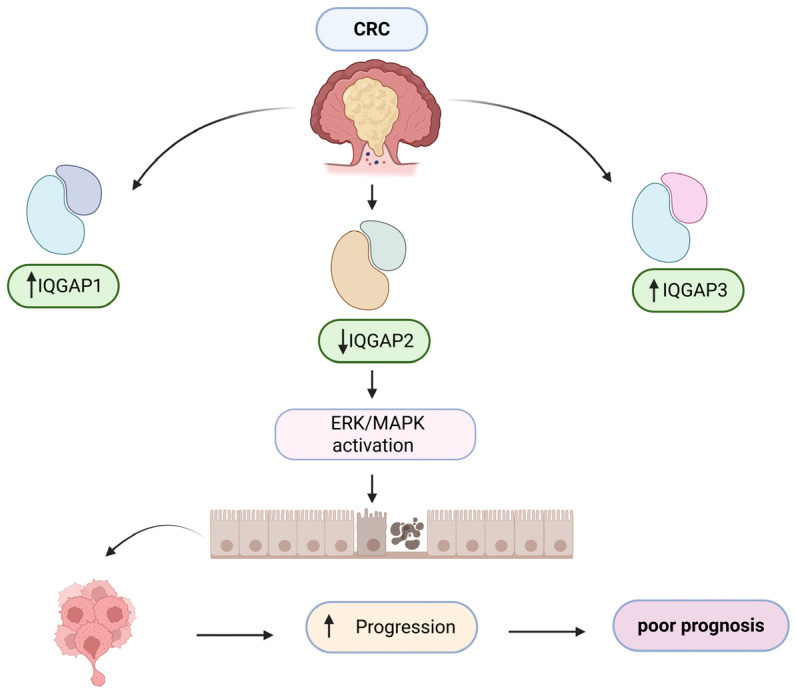
Conceptual overview of IQGAP family dysregulation in CRC. IQGAP1 and IQGAP3 are upregulated, while IQGAP2 is downregulated. Loss of IQGAP2 releases its inhibitory control over the ERK/MAPK signaling pathway, leading to sustained pathway activation. This dysregulation disrupts normal colonic epithelial architecture and promotes the emergence of malignant cell populations, ultimately driving tumor progression and contributing to poor clinical prognosis. The ↑ and ↓ symbols next to a label denote an increase or decrease, respectively, in the expression, activity, or level of that entity in CRC. Arrows indicate the direction and sequence of the depicted cascade—from IQGAP dysregulation through ERK/MAPK activation to tumor progression and poor prognosis—and do not represent expression changes. Box colors are used only to group elements for visual clarity and do not carry independent meaning.

The three human IQGAPs (IQGAP1, IQGAP2, and IQGAP3) share a similar multidomain composition, each containing a calponin homology domain (CHD), a WW domain, an IQ domain containing several IQ motifs, a GTPase-activating protein (GAP)-related domain (GRD), and a RasGAP C-terminal domain [18,50]. IQGAPs are predominantly found in the cytosol but also function in the plasma membrane to regulate receptor signaling and cell adhesion, in trafficking vesicles to coordinate endocytosis and exocytosis, and in the nucleus to influence transcription [51,52,53]. All three proteins share common binding partners, including actin, the Ca^2+^-binding protein calmodulin, and the Rho GTPases Rac1 and Cdc42. However, despite their conserved multidomain architecture, subtle differences in amino acid sequence and domain conformation among IQGAP family members result in distinct binding affinities and interaction profiles. These structural variations contribute to their differential functional roles in various cellular contexts. Furthermore, IQGAP1 is ubiquitously expressed, whereas IQGAP2 and IQGAP3 are predominantly expressed in the liver and brain, respectively [18,54]. The differential expression patterns and contrasting functional roles of the three IQGAP isoforms in CRC are summarized in Table 1, while a conceptual overview of how their dysregulation drives tumor progression and poor prognosis is illustrated in Figure 1.

## 4. IQGAP1 in Colorectal Cancer Progression and Prognosis

### 4.1. IQGAP1 Expression in CRC

IQGAP1 is the most extensively characterized member of the IQGAP protein family and has emerged as a key regulator in CRC progression [46]. Increasing evidence indicates that IQGAP1 expression is consistently elevated in CRC tissues compared to adjacent normal mucosa, suggesting its involvement in tumor development and disease progression [55]. Rather than serving as a passive marker, this upregulation appears to be functionally relevant, as higher IQGAP1 levels have been associated with enhanced cellular proliferation, invasion, and migratory capacity in CRC models [27,46,56].

At the molecular level, the functional significance of IQGAP1 overexpression is closely linked to its role as a scaffold protein capable of coordinating multiple signaling and structural components within the cell. Although initially proposed to function as a GTPase-activating protein (GAP), subsequent studies demonstrated that IQGAP1 does not promote GTP hydrolysis. Instead, it inhibits the intrinsic GTPase activity of small GTPases such as Rac1 and Cdc42, thereby stabilizing them in their active, GTP-bound states [48,57]. This sustained activation promotes actin cytoskeletal reorganization, enabling dynamic changes in cell shape that facilitate migration and invasion [19].

Structurally, IQGAP1 contains multiple domains—including the calponin homology domain (CHD), WW domain, IQ motifs, RasGAP-related domain (GRD), and RasGAP C-terminal domain (RGCT)—each of which mediates interactions with specific binding partners [46]. Through these domains, IQGAP1 binds F-actin, calmodulin, and Rho family GTPases, integrating cytoskeletal dynamics with intracellular signaling pathways. Importantly, this multidomain architecture allows IQGAP1 to function as a central platform where mechanical and biochemical processes converge [46]. The overexpression of IQGAP1 in CRC therefore reflects a shift toward a cellular state characterized by enhanced signaling integration and cytoskeletal plasticity. By stabilizing active GTPases and coordinating multiple signaling inputs, IQGAP1 promotes sustained proliferation, increased motility, and invasive behavior—processes that are functionally interconnected and position IQGAP1 as a key determinant of aggressive tumor phenotypes [17].

### 4.2. IQGAP1-Mediated ERK/MAPK Signaling

Building on its role as a structural and functional integrator, IQGAP1 is closely involved in the regulation of multiple signaling pathways that drive CRC progression. Rather than acting within a single linear cascade, IQGAP1 functions as a nodal scaffold that enables crosstalk between pathways governing proliferation, survival, and cellular motility.

A central axis within this network is the ERK/MAPK pathway. IQGAP1 interacts with key components of the RAF–MEK–ERK cascade, facilitating their spatial organization and promoting efficient signal transmission. By bringing these kinases into close proximity, IQGAP1 enhances both the efficiency and duration of ERK activation following growth factor stimulation [58,59]. Once activated, ERK translocates to the nucleus, where it phosphorylates transcription factors such as AP-1 and ELK-1. These transcription factors regulate the expression of genes involved in cell-cycle progression, including cyclin D1, as well as genes responsible for extracellular matrix remodeling, such as matrix metalloproteinases (MMP2 and MMP9). Through this mechanism, sustained ERK activation promotes both proliferative capacity and invasive behavior [60,61]. Importantly, IQGAP1-mediated scaffolding does not merely initiate ERK signaling but prolongs its activation beyond normal physiological limits. This sustained signaling state enables tumor cells to maintain growth under stress conditions while simultaneously acquiring invasive characteristics, with ERK signaling becoming tightly coupled with cytoskeletal dynamics and microRNA-mediated regulation, forming an integrated signaling network rather than an isolated pathway [62].

In addition to its role in ERK signaling, IQGAP1 also facilitates crosstalk with the Wnt/β-catenin pathway. IQGAP1 interacts with β-catenin, contributing to its stabilization and promoting its nuclear translocation. Within the nucleus, β-catenin activates transcription of genes associated with proliferation, stemness, and survival. The convergence of ERK and β-catenin signaling amplifies oncogenic outputs, reinforcing tumor progression and contributing to aggressive disease phenotypes [47].

### 4.3. Regulation of IQGAP1 by MicroRNAs

IQGAP1 expression is further regulated at the post-transcriptional level by microRNAs, particularly microRNA-124 (miR-124), which functions as a tumor suppressor in CRC. Under normal conditions, miR-124 binds to the 3′ untranslated region (3′UTR) of IQGAP1 mRNA, leading to mRNA degradation or translational repression, thereby maintaining controlled IQGAP1 expression [63,64]. In CRC, miR-124 is frequently downregulated, resulting in the loss of this regulatory checkpoint. Consequently, IQGAP1 expression increases, leading to enhanced activation of downstream signaling pathways. This upregulation amplifies ERK signaling, which, in turn, promotes transcription of genes involved in proliferation, survival, and invasion [65,66]. In parallel, increased IQGAP1 expression also enhances β-catenin signaling, further reinforcing oncogenic transcriptional programs. The combined activation of ERK and β-catenin pathways creates a synergistic effect that accelerates tumor progression. Through this mechanism, microRNA-mediated regulation converges with scaffold-dependent signaling, further highlighting the integrated nature of IQGAP1-driven oncogenic networks [67,68,69]. This regulatory axis illustrates how microRNA dysregulation can influence multiple signaling pathways through a single molecular target. Loss of miR-124 therefore represents a critical event that shifts cellular signaling toward sustained oncogenic activation and contributes to the development of aggressive tumor phenotypes [70].

### 4.4. Interaction of IQGAP1 with PLS1

The oncogenic functions of IQGAP1 are further reinforced through its interaction with cytoskeletal regulators, particularly plastin 1 (PLS1). PLS1 is an actin-bundling protein that plays a key role in organizing filamentous actin and facilitating structural rearrangements required for cell movement [71]. IQGAP1 binds to actin filaments via its calponin homology domain while simultaneously coordinating signaling molecules involved in ERK activation. PLS1 stabilizes actin bundles, thereby providing a structural framework that supports efficient signal propagation. Through this interaction, cytoskeletal organization is directly coupled to intracellular signaling pathways [72,73]. Activation of the IQGAP1–PLS1 axis enhances ERK signaling, leading to increased expression of matrix metalloproteinases, particularly MMP2 and MMP9. These enzymes degrade components of the extracellular matrix, enabling tumor cells to invade surrounding tissues and migrate to distant sites [71].

This interaction underscores the integration between mechanical and biochemical processes within cancer cells. By linking cytoskeletal remodeling to transcriptional regulation, the IQGAP1–PLS1 axis enables coordinated control of cell movement and invasive behavior, highlighting its critical role in metastatic progression.

### 4.5. Role of IQGAP1 in the Tumor Microenvironment

The tumor microenvironment (TME) plays a pivotal role in colorectal cancer progression, characterized by dynamic interactions between tumor cells and surrounding stromal components, including cancer-associated fibroblasts, immune cells, and extracellular matrix elements. This bidirectional crosstalk critically regulates tumor growth, invasion, and immune evasion by shaping both cellular behavior and signaling networks. Increasing evidence suggests that proteins involved in coordinating intracellular signaling may also influence these tumor–stromal interactions, thereby linking tumor cell-intrinsic pathways to broader microenvironmental remodeling.

In line with this, IQGAP1 extends its role beyond intracellular signaling and contributes to tumor progression through its involvement in the TME. Its interaction with molecules such as myosin light chain 9 (MYL9) suggests a role in modulating both cytoskeletal dynamics and intercellular communication [55]. MYL9 is associated with actomyosin contractility and is frequently expressed in cancer-associated fibroblasts (CAFs), which are key components of the TME. Through its interaction with IQGAP1, MYL9 may influence signaling pathways that regulate cytokine secretion and extracellular matrix remodeling [55,74]. IQGAP1-mediated signaling promotes the release of chemokines such as CCL2 and growth factors including TGF-β1, which contribute to immune cell recruitment, fibroblast activation, and extracellular matrix deposition. These processes collectively establish a pro-inflammatory and pro-fibrotic microenvironment that supports tumor growth, facilitates invasion, and promotes immune evasion [25].

This highlights an expanded role for IQGAP1 beyond intracellular signaling. By coordinating interactions between tumor cells and stromal components, IQGAP1 contributes to the dynamic remodeling of the tumor microenvironment, further amplifying disease progression. However, current evidence regarding the involvement of IQGAP2 and IQGAP3 in tumor microenvironment regulation remains limited compared with IQGAP1, highlighting an important area for future investigations.

## 5. IQGAP2 in Colorectal Cancer Progression and Prognosis

Despite belonging to the same protein family and sharing highly conserved structural domains, IQGAP1 and IQGAP2 exert opposing functional roles in CRC. This apparent paradox underscores that protein function is not solely determined by domain composition, but rather by how these domains are utilized within specific molecular and signaling contexts.

At the mechanistic level, both IQGAP1 and IQGAP2 interact with actin filaments and small GTPases such as Rac1 and Cdc42; however, the downstream consequences of these interactions diverge significantly [75,76]. IQGAP1 functions as an efficient scaffold that promotes the assembly of signaling complexes, particularly within the ERK/MAPK pathway, thereby facilitating sustained signal transduction and oncogenic output [77]. In contrast, IQGAP2 appears to regulate these same signaling networks through mechanisms that limit signal propagation. Although the precise molecular mechanisms remain incompletely defined, IQGAP2 may interfere with scaffold formation, compete with IQGAP1 for shared binding partners, and regulate the spatial organization of signaling platforms [78]. Through these mechanisms, IQGAP2 attenuates ERK activation and prevents excessive amplification of oncogenic signaling [28,29].

The opposing functions of IQGAP1 and IQGAP2 are further reinforced by their differential expression in CRC, where IQGAP1 is frequently upregulated, while IQGAP2 is commonly downregulated. This imbalance shifts intracellular signaling toward sustained activation, highlighting the importance of IQGAP family balance in regulating tumor behavior.

### 5.1. IQGAP2 Expression in CRC

IQGAP2 expression is consistently reduced in CRC tissues compared to adjacent normal mucosa, suggesting its role as a tumor suppressor. This downregulation has been observed across multiple gastrointestinal malignancies, indicating that loss of IQGAP2 may represent a common event in epithelial tumorigenesis [79].

One of the primary mechanisms underlying reduced IQGAP2 expression is epigenetic silencing, particularly through promoter hypermethylation [80,81]. This modification leads to decreased transcriptional activity, thereby reducing protein expression levels [82]. In addition to epigenetic regulation, IQGAP2 expression may also be influenced by transcriptional and post-transcriptional mechanisms, including microRNA-mediated regulation, reflecting the complex regulatory landscape governing IQGAP2 expression [79,83].

Importantly, reduced IQGAP2 expression is not merely a bystander effect but appears to confer a selective advantage to tumor cells. Cells with diminished IQGAP2 levels exhibit increased proliferative and invasive capabilities, suggesting that loss of IQGAP2 contributes directly to tumor progression [31]. This is further supported by the observed inverse relationship between IQGAP1 and IQGAP2 expression, where increased IQGAP1 levels coincide with reduced IQGAP2 expression, reinforcing a shift toward oncogenic signaling dominance [84].

### 5.2. Regulation of IQGAP2 by miR-29a

In addition to epigenetic silencing, IQGAP2 is subject to post-transcriptional regulation by microRNAs, particularly miR-29a-3p, which has been implicated in CRC progression. Dysregulation of miR-29a is frequently observed in CRC and is associated with enhanced tumor growth, invasion, and metastasis [85,86,87]. Mechanistically, miR-29a-3p directly binds to the 3′-UTR of IQGAP2 mRNA, leading to mRNA degradation or inhibition of translation. This results in decreased IQGAP2 protein levels, thereby weakening its role as a negative regulator of oncogenic signaling pathways. The loss of this regulatory constraint facilitates increased activation of downstream pathways involved in cell proliferation and migration [88,89].

Furthermore, the miR-29a–IQGAP2 axis illustrates how non-coding RNAs integrate into broader signaling networks. By targeting a scaffold protein such as IQGAP2, miR-29a indirectly modulates multiple downstream pathways simultaneously, amplifying its impact on tumor biology. Restoration of IQGAP2 expression in experimental models has been shown to partially reverse the pro-tumorigenic effects of miR-29a, further supporting this regulatory relationship [80].

### 5.3. IQGAP2 and ERK/MAPK Signaling Suppression

A key mechanism underlying the tumor-suppressive function of IQGAP2 involves its modulation of the ERK/MAPK signaling pathway, a central regulator of colorectal cancer progression. While IQGAP1 enhances ERK/MAPK signaling by facilitating the assembly of kinase complexes, IQGAP2 exerts an opposing effect by attenuating ERK activation [29].

This suppression may occur through multiple mechanisms. IQGAP2 may interfere with the assembly or stability of signaling complexes required for efficient ERK phosphorylation, thereby reducing signaling efficiency. Additionally, it may compete with IQGAP1 for shared binding partners, limiting the formation of pro-oncogenic signaling platforms. IQGAP2 may also influence the spatial and temporal dynamics of signaling, restricting the localization and duration of ERK/MAPK activation [89]. Through these mechanisms, IQGAP2 reduces the expression of downstream genes involved in proliferation, migration, and extracellular matrix remodeling. This regulatory effect highlights its role not only as a structural scaffold but also as a modulator of signaling intensity and duration. The balance between IQGAP1-mediated activation and IQGAP2-mediated suppression is therefore critical in determining overall signaling output in CRC cells.

Nevertheless, the available evidence regarding IQGAP2-mediated regulation of ERK/MAPK signaling in CRC remains relatively limited, highlighting the need for further investigation to elucidate its precise molecular mechanisms and potential therapeutic implications.

### 5.4. Prognostic Relevance of IQGAP2

At the cellular level, reduced IQGAP2 expression is associated with increased motility and invasive capacity, key features of cancer progression [89]. These effects are closely linked to alterations in cytoskeletal organization and cell adhesion. Notably, decreased IQGAP2 expression has been associated with reduced E-cadherin levels, a hallmark of epithelial–mesenchymal transition (EMT) [90]. As a key component of adherens junctions, E-cadherin plays a critical role in maintaining epithelial integrity [91]. Its loss facilitates cellular detachment, increased motility, and acquisition of mesenchymal characteristics, thereby promoting metastasis. IQGAP2 may contribute to the stabilization of cytoskeletal architecture and cell–cell adhesion, and its loss disrupts these processes, favoring EMT [92].

Clinically, these molecular changes are reflected in more aggressive tumor phenotypes, where low IQGAP2 expression has been associated with advanced tumor stage, higher histological grade, and poorer survival outcomes [93]. These findings suggest that IQGAP2 may serve as a potential prognostic biomarker in CRC; however, further large-scale clinical studies are required to validate its prognostic value and determine its potential role in therapeutic targeting.

Taken together, IQGAP2 functions as a key regulator of signaling restraint in colorectal cancer, modulating both intracellular signaling pathways and cytoskeletal dynamics. Its loss removes a critical inhibitory checkpoint, promoting oncogenic signaling, cellular plasticity, and tumor progression. Importantly, the balance between IQGAP2 and its oncogenic counterparts, particularly IQGAP1, plays a central role in determining disease behavior, highlighting the IQGAP family as an integrated regulatory network.

## 6. Oncogenic Role of IQGAP3 in CRC

While IQGAP1 and IQGAP2 have been relatively well characterized as an oncogenic signal amplifier and a tumor suppressor, respectively, the role of IQGAP3 in CRC reflects a distinct functional specialization within the IQGAP family. Despite sharing a conserved structural framework, these proteins exhibit divergent biological functions, indicating that their roles are determined less by domain composition and more by differences in binding partners, signaling context, and spatial regulation [94]. In contrast to IQGAP1, which predominantly enhances proliferative signaling through ERK/MAPK pathways, and IQGAP2, which restrains signaling and maintains cellular stability, IQGAP3 appears to preferentially amplify signaling processes that couple cytoskeletal remodeling with cellular motility [95]. This positioning suggests that IQGAP3 plays a central role in coordinating the transition from intracellular signaling activation to invasive cellular behavior [30,96].

### 6.1. Expression and Clinical Correlation of IQGAP3

IQGAP3 has consistently been reported to be upregulated in CRC, with elevated expression observed in tumor tissues relative to adjacent normal mucosa [97,98]. Importantly, this upregulation is closely associated with clinicopathological features indicative of aggressive disease, including advanced tumor–node–metastasis (TNM) stage, lymph node involvement, vascular invasion, and poor tumor differentiation. In addition, increased IQGAP3 expression is associated with reduced overall survival, suggesting that its expression reflects not only tumor presence but also the biological aggressiveness of the disease [98].

The functional relevance of this upregulation becomes evident when considered in the context of IQGAP3’s role as a scaffold protein. Scaffold proteins regulate signaling by organizing multiple signaling components into coordinated complexes, thereby controlling both the efficiency and specificity of signal transduction [99,100]. In this setting, overexpression of IQGAP3 increases the density of interaction platforms within the cell, facilitating the assembly of signaling complexes and enhancing the likelihood of productive protein–protein interactions.

This increase in scaffolding capacity has two major consequences. First, it enhances signaling efficiency by reducing the spatial separation between pathway components, allowing for rapid and coordinated signal transmission. Second, it prolongs signal duration by stabilizing signaling complexes and preventing premature dissociation. Together, these effects shift intracellular signaling from transient, tightly regulated activation toward sustained and amplified signaling output [44,94]. Such sustained signaling is a hallmark of oncogenic transformation and provides a mechanistic explanation for the association between high IQGAP3 expression and aggressive tumor behavior. In this context, IQGAP3 overexpression does not merely accompany tumor progression but actively contributes to the establishment of a signaling environment that favors persistent activation of pathways involved in survival, motility, and invasion.

### 6.2. IQGAP3 and Oncogenic Signaling Pathways

The oncogenic role of IQGAP3 is primarily mediated through its ability to coordinate intracellular signaling pathways via its scaffolding function. Rather than acting within a single linear pathway, IQGAP3 functions as a central organizer that integrates and amplifies multiple signaling inputs, thereby increasing overall signaling output.

Among the pathways associated with IQGAP3, the phosphoinositide 3-kinase (PI3K) pathway represents a critical axis. IQGAP3 facilitates the assembly of signaling complexes involving PI3K components, including PIK3C2B, thereby enhancing pathway activation. This process is driven by spatial organization, as IQGAP3 brings upstream activators, PI3K components, and downstream effectors into close proximity, enabling efficient and coordinated signal propagation [101,102].

Activation of PI3K signaling initiates a multi-step cascade that links intracellular signaling to cellular behavior. One key branch involves activation of Rho family GTPases, including Rac1 and Cdc42, which regulate actin cytoskeleton dynamics. IQGAP3 likely facilitates localized activation of these GTPases at specific cellular regions, particularly at the plasma membrane and leading edge, where cytoskeletal remodeling is required for directional movement [103,104,105].

This spatial regulation is critical for effective cell migration. Activation of Rac1 promotes actin polymerization and the formation of lamellipodia, enabling forward membrane extension and generating traction forces required for movement. In parallel, activation of Cdc42 induces filopodia formation, which allows cells to sense environmental cues and guide directional migration. The coordinated activity of these structures enables tumor cells to polarize and migrate through complex tissue environments [106]. At the same time, PI3K signaling activates downstream survival pathways, including those mediated by AKT, which enhance resistance to apoptosis [107]. This is particularly important during tumor dissemination, where cells must survive under conditions of detachment and environmental stress. The simultaneous activation of cytoskeletal remodeling and survival signaling creates a cellular state that is highly conducive to invasion and metastasis [108].

Importantly, IQGAP3 operates within a broader signaling network that may include interactions with growth factor–mediated pathways such as EGFR and ERK/MAPK [109]. However, unlike IQGAP1, which predominantly amplifies proliferative ERK signaling, IQGAP3 appears to preferentially engage signaling pathways that link cytoskeletal dynamics with cellular motility. This functional specialization underscores the distinct role of IQGAP3 within the IQGAP family.

### 6.3. Role of IQGAP3 in Tumor Progression and Cellular Dynamics

The signaling processes coordinated by IQGAP3 are ultimately translated into changes in cellular behavior that drive tumor progression. IQGAP3 plays a central role in regulating cell migration, invasion, and structural plasticity, processes that are essential for cancer dissemination [110].

Through its effects on Rho GTPase activation and actin cytoskeleton remodeling, IQGAP3 enables tumor cells to undergo dynamic morphological changes required for movement. The formation of lamellipodia allows cells to extend their membrane forward and generate traction forces necessary for migration, while filopodia provide directional sensing that guides movement through the extracellular matrix [111,112]. These coordinated processes enable tumor cells to detach from the primary tumor mass and invade surrounding tissues.

Importantly, cytoskeletal remodeling alone is insufficient for successful invasion. Tumor cells must also survive during detachment and migration, processes that would normally induce apoptosis through anoikis. However, activation of PI3K-related survival pathways enables tumor cells to resist these signals, allowing them to persist in non-adherent conditions and continue the metastatic process [113]. The integration of increased motility and enhanced survival results in a highly adaptive cellular phenotype characterized by increased plasticity. This phenotype shares key features with epithelial–mesenchymal transition (EMT), including reduced cell–cell adhesion, increased migratory capacity, and enhanced invasiveness. Although the precise involvement of IQGAP3 in EMT-associated transcriptional regulation remains to be fully defined, its ability to coordinate signaling and cytoskeletal dynamics strongly supports its role in promoting EMT-like behavior [94].

### 6.4. Prognostic Significance of IQGAP3

The molecular and cellular functions of IQGAP3 are closely reflected in its clinical significance. Elevated IQGAP3 expression is consistently associated with poorer patient outcomes, including reduced overall survival, indicating that it serves as a marker of aggressive disease [86].

Mechanistically, the prognostic impact of IQGAP3 can be understood as a direct consequence of its role in coordinating signaling pathways that promote both invasion and survival. By enhancing PI3K-related signaling and cytoskeletal remodeling, IQGAP3 facilitates tumor cell dissemination, while its effects on survival pathways enable cells to persist during metastasis. These combined effects increase the likelihood of disease progression and contribute to poorer clinical outcomes [94].

Importantly, IQGAP3 does not function in isolation but operates within an interconnected signaling network. Tumors with high IQGAP3 expression often exhibit concurrent activation of PI3K and cytoskeleton-regulating pathways, resulting in a more aggressive phenotype. This network-based effect amplifies its impact on tumor progression and highlights the importance of considering signaling context in prognostic assessment. Furthermore, the prognostic significance of IQGAP3 may be influenced by the broader balance within the IQGAP family. While IQGAP2 acts to restrain signaling and maintain cellular stability, its downregulation removes this regulatory constraint. In the presence of elevated IQGAP3, this imbalance shifts the cellular environment toward sustained oncogenic signaling, enhanced motility, and increased invasive capacity. This coordinated dysregulation further contributes to poor clinical outcomes [97,98].

Taken together, these observations position IQGAP3 as both a marker and a driver of aggressive disease. Its ability to integrate signaling pathways that regulate invasion, survival, and cellular plasticity provides a mechanistic basis for its strong association with adverse prognosis, highlighting its potential as a prognostic biomarker and therapeutic target in colorectal cancer.

## 7. Clinical and Prognostic Implications of IQGAP Family Proteins

Throughout this section, the terms diagnostic biomarker, prognostic indicator, and therapeutic target are used to denote distinct clinical concepts and are addressed separately. A diagnostic biomarker supports the detection or classification of disease, a prognostic indicator informs the prediction of clinical outcome, and a therapeutic target denotes a molecule amenable to therapeutic intervention. Accordingly, the potential roles of IQGAP proteins in each of these domains are considered individually in the following subsections, and the strength of the available evidence differs across these applications.

### 7.1. Biomarker Potential

The distinct and opposing roles of IQGAP family members in CRC provide a strong rationale for their consideration as potential biomarkers. Unlike conventional single-pathway markers, IQGAP proteins function as integrators of intracellular signaling, coordinating multiple oncogenic pathways within a unified regulatory framework. As a result, alterations in their expression reflect shifts in overall signaling dynamics rather than isolated molecular changes [114].

In CRC, differential expression patterns of IQGAP family members are closely associated with tumor behavior. IQGAP1 is associated with enhanced proliferative capacity, while IQGAP3 is associated with enhanced invasive capacity. IQGAP2 is frequently downregulated, indicating loss of regulatory control over these processes. This coordinated dysregulation suggests that IQGAP expression profiles represent the functional balance between signaling activation and inhibition within tumor cells [115].

The biomarker potential of IQGAP proteins arises from their involvement in key steps of tumor progression. IQGAP1 contributes to early stages of the metastatic cascade, including invasion and extravasation, through its regulation of actin dynamics and MAPK signaling. In parallel, IQGAP3 facilitates the integration of signaling pathways that promote cytoskeletal remodeling and survival, enabling tumor cells to migrate and persist under stress conditions. These roles highlight how IQGAP proteins translate signaling activity into invasive cellular behavior [27].

Importantly, this integrative function distinguishes IQGAP proteins from conventional biomarkers that typically reflect single molecular alterations. Tumor progression is driven by coordinated signaling networks rather than isolated pathways; therefore, biomarkers that capture these network-level changes may provide a more functionally relevant representation of tumor behavior. In this context, IQGAP proteins offer potential as integrative biomarkers that reflect the dynamic state of signaling activity underlying CRC progression.

### 7.2. Prognostic Indicator

Beyond their role as biomarkers, IQGAP family proteins also demonstrate significant value as prognostic indicators in CRC, as their expression levels are closely associated with disease progression and clinical outcomes. Importantly, the prognostic relevance of IQGAP proteins is not merely based on statistical associations but reflects their direct involvement in key biological processes that drive tumor aggressiveness [79,116].

Elevated expression of IQGAP1 and IQGAP3, along with reduced expression of IQGAP2, is consistently associated with poorer clinical outcomes. This pattern reflects a shift in the balance of intracellular signaling toward sustained oncogenic activation. IQGAP1 promotes persistent activation of proliferative pathways, particularly through ERK/MAPK signaling, leading to increased tumor growth [117]. In parallel, IQGAP3 enhances signaling pathways that regulate cytoskeletal remodeling and cellular motility, thereby facilitating invasion and metastatic dissemination [30]. Conversely, downregulation of IQGAP2 removes critical inhibitory constraints on these processes, further amplifying oncogenic signaling [86].

This coordinated dysregulation contributes to multiple hallmarks of aggressive disease. Increased proliferative signaling promotes tumor expansion, while enhanced cytoskeletal dynamics enable tumor cells to invade surrounding tissues and migrate to distant sites. At the same time, activation of survival pathways allows tumor cells to resist apoptosis, particularly during detachment and metastasis [118]. The integration of these processes results in a highly adaptive cellular phenotype characterized by increased plasticity and metastatic potential. These biological effects translate into poorer patient outcomes, including reduced overall survival and increased likelihood of disease progression. Importantly, the prognostic value of IQGAP proteins is further enhanced when considered in combination rather than in isolation. For example, tumors characterized by high IQGAP3 expression together with reduced IQGAP2 expression may represent a particularly high-risk subgroup, reflecting both increased signaling activation and loss of regulatory control [79].

Taken together, these findings suggest that IQGAP proteins serve as functional prognostic indicators that capture the dynamic interplay between signaling activation, cellular behavior, and clinical outcome. By linking molecular mechanisms to disease progression, IQGAP expression profiles provide a biologically meaningful framework for predicting prognosis in CRC.

### 7.3. Therapeutic Target

The RTK–RAS–RAF–MEK–ERK signaling pathway is a central driver of tumor progression and a major target of current cancer therapies [119]. However, despite the development of inhibitors targeting multiple components of this cascade, therapeutic efficacy remains limited by the complexity and adaptability of signaling networks. In this context, increasing attention has shifted toward regulatory proteins that organize and sustain signaling activity, including scaffold proteins such as the IQGAP family [114].

Scaffold proteins regulate signaling not by initiating enzymatic reactions, but by organizing signaling components into coordinated complexes. Through this function, they enhance both the efficiency and specificity of signal transduction by facilitating protein–protein interactions and stabilizing signaling assemblies. Targeting scaffold-mediated interactions therefore offers a distinct therapeutic strategy, as it may disrupt multiple downstream pathways simultaneously rather than inhibiting a single catalytic step [114].

Within this framework, IQGAP family proteins have emerged as potential therapeutic targets due to their central role in coordinating oncogenic signaling. IQGAP1, the most extensively studied member, interacts with key components of the MAPK pathway, including RAF, MEK, and ERK, thereby promoting sustained proliferative signaling. In addition, its role in cytoskeletal organization and cell–cell adhesion links signaling activity to migration and invasion, further contributing to tumor progression [120].

Other members of the IQGAP family contribute to distinct aspects of tumor behavior. IQGAP3 is associated with signaling pathways that regulate cytoskeletal remodeling and cellular motility, suggesting that targeting its scaffolding function may impair tumor invasion and metastatic dissemination [30]. In contrast, IQGAP2 exhibits tumor-suppressive properties, and its loss contributes to enhanced signaling activity and disease progression. These complementary roles highlight the potential of targeting the IQGAP family as a means of modulating signaling balance at a network level [29].

Despite these promising features, targeting scaffold proteins presents unique challenges. Unlike kinases, scaffold proteins lack intrinsic catalytic activity, making them less amenable to traditional small-molecule inhibition. As a result, therapeutic strategies must focus on disrupting protein–protein interactions or modulating scaffold protein expression [121]. Alternatively, targeting downstream pathways regulated by IQGAP proteins may provide an indirect means of counteracting their effects.

Taken together, IQGAP family proteins represent a novel class of therapeutic targets that operate at the level of signaling organization rather than individual pathway components. By modulating scaffold-mediated interactions, it may be possible to disrupt multiple oncogenic pathways simultaneously, offering a potential strategy to overcome the limitations of current targeted therapies in colorectal cancer.

Currently, evidence supporting IQGAP proteins as diagnostic biomarkers is largely derived from retrospective expression analyses and experimental studies. Standardized immunohistochemical scoring systems, transcriptomic cut-off values, and prospective validation cohorts are still lacking. Furthermore, it remains unclear whether IQGAP expression provides independent prognostic value beyond established CRC markers such as TNM stage, MSI/MMR status, and KRAS/NRAS/BRAF alterations.

### 7.4. Precision Oncology

The integration of molecular profiling into clinical decision-making has shifted the focus of cancer management toward precision oncology, where treatment strategies are tailored based on the biological characteristics of individual tumors [122]. In this context, understanding not only the presence of specific molecular alterations but also the functional state of signaling networks becomes increasingly important.

IQGAP family proteins may provide a unique perspective within this framework for understanding signaling integration, as they function at the level of signaling integration rather than individual pathway components. Their expression reflects the dynamic coordination of multiple oncogenic processes, including proliferation, cytoskeletal remodeling, and cellular survival [18]. As such, IQGAP proteins offer insight into how signaling pathways operate collectively within tumor cells, rather than in isolation.

This network-level perspective may have important implications for patient stratification. Tumors characterized by distinct patterns of IQGAP expression patterns, such as increased IQGAP1 and IQGAP3, alongside reduced IQGAP2, may represent a subgroup with enhanced signaling activity, increased invasive potential, and a higher likelihood of disease progression. Identifying such patterns could support more refined risk classification and guide treatment selection.

In addition, the integration of IQGAP expression profiles with other molecular data, including genomic and transcriptomic information, may further improve the ability to predict therapeutic response. Rather than relying on single-gene alterations, this approach emphasizes the importance of signaling context, where the effectiveness of targeted therapies may depend on the broader regulatory environment within the tumor [18].

**Figure 2:**
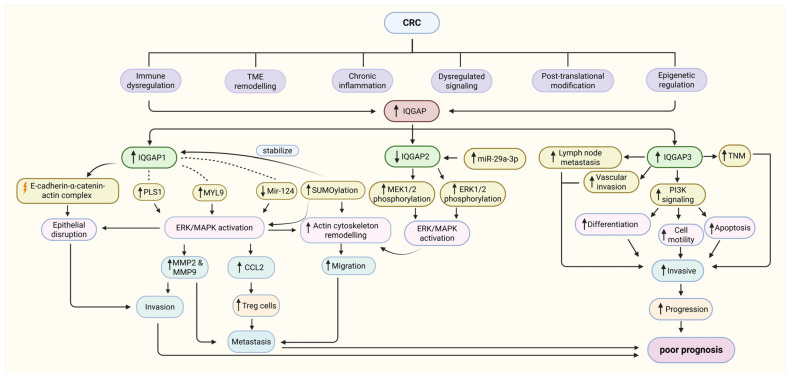
Integrated overview of the upstream regulators, isoform-specific molecular pathways, and downstream cellular and clinical consequences of IQGAP family dysregulation in colorectal cancer (CRC). Multiple factors, including immune dysregulation, tumor microenvironment remodeling, chronic inflammation, dysregulated signaling, post-translational modifications, and epigenetic regulation, influence IQGAP family expression and activity. Differential alterations in IQGAP1, IQGAP2, and IQGAP3 modulate key signaling pathways involved in proliferation, cytoskeletal remodeling, invasion, metastasis, and tumor progression, ultimately contributing to adverse clinical outcomes in CRC. The figure is organized into three stages: factors influencing IQGAP family expression and activity (left), isoform-specific signaling by IQGAP1, IQGAP2, and IQGAP3 (center), and the resulting cellular and clinical consequences (right). Upward (↑) and downward (↓) arrows indicate an increase or decrease, respectively, in the expression, activity, or level of the labeled molecule or process in CRC. Solid arrows represent a promoting or directional effect—activation, downstream signaling, or progression—between connected elements. Dashed lines denote interaction or regulatory relationships between IQGAP1 and its molecular partners (PLS1, MYL9, and miR-124). Box colors are used only to group elements by category for visual clarity and do not carry independent meaning; the three IQGAP isoforms are identified by their labels rather than by color.

These considerations highlight the potential role of IQGAP proteins in bridging molecular characterization with functional tumor behavior. By capturing the interplay between signaling pathways and cellular dynamics, IQGAP-based profiling may contribute to more precise and biologically informed treatment strategies in CRC. A comprehensive integrated overview of the upstream drivers, isoform-specific molecular pathways, and downstream cellular and clinical consequences of IQGAP dysregulation in CRC discussed throughout this review is presented in Figure 2.

## 8. Future Perspectives

Despite growing evidence supporting the role of IQGAP family proteins in CRC progression, several key questions remain unresolved, highlighting important directions for future research. Much of the current understanding is derived from in vitro studies and limited clinical datasets, and further validation in larger, well-characterized patient cohorts is necessary to establish the clinical utility of IQGAP proteins as biomarkers and therapeutic targets. Furthermore, several mechanistic insights discussed in this review are derived from studies conducted in other malignancies, including breast, ovarian, gastric, and hepatocellular cancers. Although these findings provide valuable biological insights, their applicability to CRC requires further validation in CRC-specific experimental models and clinical cohorts.

One important area for future investigation is the precise molecular mechanisms underlying the differential functions of IQGAP family members. Although IQGAP1 and IQGAP3 are generally associated with oncogenic signaling, while IQGAP2 exhibits tumor-suppressive properties, the factors that determine these functional differences remain incompletely understood [123]. In particular, the role of context-dependent interactions, post-translational modifications, and subcellular localization in shaping IQGAP activity warrants further exploration. A more detailed understanding of these mechanisms may clarify how structurally similar proteins exert distinct biological effects within the same cellular environment.

In addition, the interplay between IQGAP family members within shared signaling networks represents an important but underexplored area. Rather than acting independently, IQGAP proteins may function as part of a coordinated regulatory system in which the balance between signal amplification and restraint determines overall cellular behavior. Investigating how alterations in the relative expression of IQGAP1, IQGAP2, and IQGAP3 influence signaling dynamics may provide deeper insight into CRC progression and identify novel points of therapeutic intervention.

From a translational perspective, further work is needed to evaluate the feasibility of targeting IQGAP-mediated signaling in clinical settings. Given the challenges associated with directly inhibiting scaffold proteins, future studies should explore strategies aimed at disrupting protein–protein interactions or modulating downstream signaling pathways. Advances in drug development, including the design of molecules that specifically interfere with scaffold interactions, may offer new opportunities for therapeutic intervention.

Finally, the integration of IQGAP-related findings into multi-omics approaches may enhance their clinical relevance. Combining genomic, transcriptomic, and proteomic data could provide a more comprehensive understanding of how IQGAP proteins contribute to tumor heterogeneity and treatment response. Such approaches may also support the development of more refined patient stratification models, aligning with the broader goals of precision oncology. However, standardized detection methods, validated cut-off values, and prospective clinical studies are still required before IQGAP proteins can be considered reliable diagnostic or prognostic biomarkers in routine clinical practice.

## 9. Conclusions

The IQGAP family of scaffold proteins plays a central role in regulating signaling networks that drive CRC progression. Despite their structural similarity, IQGAP1, IQGAP2, and IQGAP3 exhibit distinct and often opposing functions, with increased IQGAP1 and IQGAP3, alongside reduced IQGAP2, collectively promoting sustained oncogenic signaling, enhanced cellular plasticity, and aggressive tumor behavior.

These coordinated roles suggest that IQGAP proteins may serve as integrative biomarkers and potential therapeutic targets; however, prospective clinical validation is still required.

## Figures and Tables

**Table 1:** Differential expression and functional roles of IQGAP isoforms in colorectal cancer.

Isoform	Expression	Main Role	Key Pathways	Clinical Association	Detection Method	Prognostic Relevance
IQGAP1	Upregulated	Scaffold protein regulating signaling cascades	ERK/MAPK	Invasion, migration	IHC, mRNA	Poor overall survival
IQGAP2	Downregulated	Tumor suppressor	Wnt/β-catenin	EMT, progression	IHC, mRNA	Associated with unfavorable prognosis
IQGAP3	Upregulated	Regulator of cell proliferation	ERK, PI3K	Metastasis, EMT	IHC, mRNA	Associated with unfavorable prognosis

## Data Availability

No new data were created or analyzed in this study. Data sharing is not applicable to this article.

## Abbreviations

The following abbreviations are used in this manuscript:

3′UTR	3′ Untranslated Region
AKT	Protein Kinase B
AP-1	Activator Protein 1
CAF	Cancer-Associated Fibroblast
CCL2	C-C motif Chemokine Ligand 2
Cdc42	Cell Division Cycle 42
CHD	Calponin Homology Domain
CRC	Colorectal Cancer
EGFR	Epidermal Growth Factor Receptor
ELK-1	ETS-Like protein 1
EMT	Epithelial–Mesenchymal Transition
ERK	Extracellular signal-Regulated Kinase
F-actin	Filamentous Actin
GAP	GTPase-Activating Protein
GRD	Ras GTPase-Related Domain
GTP	Guanosine Triphosphate
IQGAP	IQ motif-containing GTPase-Activating Protein
Irs	IQGAP-specific Repeats
MAPK	Mitogen-Activated Protein Kinase
MEK	MAPK/ERK Kinase
miRNA/miR	microRNA
MMP	Matrix Metalloproteinase
mRNA	messenger RNA
MYL9	Myosin Light Chain 9
PI3K	Phosphoinositide 3-Kinase
PIK3C2B	Phosphatidylinositol-4-phosphate 3-Kinase Catalytic Subunit Type 2 Beta
PIP2	Phosphatidylinositol 4,5-Bisphosphate
PLS1	Plastin 1
Rac1	Ras-related C3 botulinum toxin substrate 1
RAF	Rapidly Accelerated Fibrosarcoma kinase
RAS	Rat Sarcoma virus oncogene homolog
RasGAP	Ras GTPase-Activating Protein
RGCT	RasGAP C-Terminal domain
RTK	Receptor Tyrosine Kinase
TGF-β	Transforming Growth Factor-beta
TME	Tumor Microenvironment
TNM	Tumor–Node–Metastasis
Wnt	Wingless-related integration site
WW	WW domain (containing two conserved tryptophan residues)

## References

[1] Abedin K., Lean Q.Y., Wheelwright S. (2024). Nutritional self-management in colorectal cancer patients and survivors: A scoping review. Colorectal Dis..

[2] Rawla P., Sunkara T., Barsouk A. (2019). Epidemiology of colorectal cancer: Incidence, mortality, survival, and risk factors. Prz. Gastroenterol..

[3] Xi Y., Xu P. (2021). Global colorectal cancer burden in 2020 and projections to 2040. Transl. Oncol..

[4] Roshandel G., Ghasemi-Kebria F., Malekzadeh R. (2024). Colorectal cancer: Epidemiology, risk factors, and prevention. Cancers.

[5] Testa U., Castelli G., Pelosi E. (2020). Genetic alterations of metastatic colorectal cancer. Biomedicines.

[6] Li Q., Geng S., Luo H., Wang W., Mo Y.Q., Luo Q., Wang L., Song G.B., Sheng J.P., Xu B. (2024). Signaling pathways involved in colorectal cancer: Pathogenesis and targeted therapy. Signal Transduct. Target. Ther..

[7] Kasi A., Handa S., Bhatti S., Umar S., Bansal A., Sun W. (2020). Molecular pathogenesis and classification of colorectal carcinoma. Curr. Colorectal Cancer Rep..

[8] Liu Y., Lau H.C.H., Cheng W.Y., Yu J. (2022). Gut microbiome in colorectal cancer: Clinical diagnosis and treatment. Genom. Proteom. Bioinform..

[9] Fares J., Fares M.Y., Khachfe H.H., Salhab H.A., Fares Y. (2020). Molecular principles of metastasis: A hallmark of cancer revisited. Signal Transduct. Target. Ther..

[10] Valdespino-Gómez V.M., Valdespino-Castillo P.M., Valdespino-Castillo V.E. (2015). Cell signalling pathways interaction in cellular proliferation: Potential target for therapeutic interventionism. Cir. Cir..

[11] Sanchez-Vega F., Mina M., Armenia J., Chatila W.K., Luna A., La K.C., Dimitriadoy S., Liu D.L., Kantheti H.S., Saghafinia S. (2018). Oncogenic signaling pathways in The Cancer Genome Atlas. Cell.

[12] Zhao H., Ming T., Tang S., Ren S., Yang H., Liu M., Tao Q., Xu H. (2022). Wnt signaling in colorectal cancer: Pathogenic role and therapeutic target. Mol. Cancer.

[13] Zhu Y., Li X. (2023). Advances of Wnt signalling pathway in colorectal cancer. Cells.

[14] Itatani Y., Kawada K., Sakai Y. (2019). Transforming growth factor-β signaling pathway in colorectal cancer and its tumor microenvironment. Int. J. Mol. Sci..

[15] Song Y.Y., Liang D., Liu D.K., Lin L., Zhang L., Yang W.Q. (2023). The role of the ERK signaling pathway in promoting angiogenesis for treating ischemic diseases. Front. Cell Dev. Biol..

[16] Xue C., Chu Q., Shi Q., Zeng Y., Lu J., Li L. (2025). Wnt signaling pathways in biology and disease: Mechanisms and therapeutic advances. Signal Transduct. Target. Ther..

[17] Smith J.M., Hedman A.C., Sacks D.B. (2015). IQGAPs choreograph cellular signaling from the membrane to the nucleus. Trends Cell Biol..

[18] Thines L., Roushar F.J., Hedman A.C., Sacks D.B. (2023). The IQGAP scaffolds: Critical nodes bridging receptor activation to cellular signaling. J. Cell Biol..

[19] Wei T., Lambert P.F. (2021). Role of IQGAP1 in carcinogenesis. Cancers.

[20] Dong P., Ihira K., Xiong Y., Watar H., Hanley S.J.B., Yamada T., Hosaka M., Kudo M., Yue J., Sakuragi N. (2016). Reactivation of epigenetically silenced miR-124 reverses the epithelial-to-mesenchymal transition and inhibits invasion in endometrial cancer cells via the direct repression of IQGAP1 expression. Oncotarget.

[21] Liu Z., Li X., Ma J., Li D.C., Ju H.X., Liu Y., Chen Y.B., He X.J., Zhu Y.P. (2020). Integrative analysis of the IQ motif-containing GTPase-activating protein family indicates that the IQGAP3-PIK3C2B axis promotes invasion in colon cancer. OncoTargets Ther..

[22] Hedman A.C., Smith J.M., Sacks D.B. (2015). The biology of IQGAP proteins: Beyond the cytoskeleton. EMBO Rep..

[23] Adachi M., Kawasaki A., Nojima H., Nishida E., Tsukita S. (2014). Involvement of IQGAP family proteins in the regulation of mammalian cell cytokinesis. Genes Cells.

[24] White C.D., Brown M.D., Sacks D.B. (2009). IQGAPs in cancer: A family of scaffold proteins underlying tumorigenesis. FEBS Lett..

[25] Morgan C.J., Hedman A.C., Li Z., Sacks D.B. (2019). Endogenous IQGAP1 and IQGAP3 do not functionally interact with Ras. Sci. Rep..

[26] Briggs M.W., Sacks D.B. (2003). IQGAP proteins are integral components of cytoskeletal regulation. EMBO Rep..

[27] Hebert J.D., Tian C., Lamar J.M., Rickelt S., Abbruzzese G., Liu X.T., Hynes R.O. (2020). The scaffold protein IQGAP1 is crucial for extravasation and metastasis. Sci. Rep..

[28] Vaitheesvaran B., Hartil K., Navare A., Zheng C., Broin P.Ó., Golden A., Guha C., Lee W.N., Kurland I.J., Bruce J.E. (2014). Role of the tumor suppressor IQGAP2 in metabolic homeostasis: Possible link between diabetes and cancer. Metabolomics.

[29] Kumar D., Patel S.A., Hassan M.K., Mohapatra N., Pattanaik N., Dixit M. (2021). Reduced IQGAP2 expression promotes EMT and inhibits apoptosis by modulating the MEK-ERK and p38 signaling in breast cancer irrespective of ER status. Cell Death Dis..

[30] Dongol S., Zhang Q., Qiu C., Sun C., Zhang Z., Wu H., Kong B. (2020). IQGAP3 promotes cancer proliferation and metastasis in high-grade serous ovarian cancer. Oncol. Lett..

[31] Liao Y., Chen X., Miller-Little W., Wang H., Williard B., Bulek K., Zhao J., Li X. (2023). The Ras GTPase-activating-like protein IQGAP1 bridges gasdermin D to the ESCRT system to promote IL-1β release via exosomes. EMBO J..

[32] Bachir A.I., Horwitz A.R., Nelson W.J., Bianchini J.M. (2017). Actin-based adhesion modules mediate cell interactions with the extracellular matrix and neighboring cells. Cold Spring Harb. Perspect. Biol..

[33] Yin L.M., Schnoor M., Jun C.D. (2020). Structural characteristics, binding partners and related diseases of the calponin homology (CH) domain. Front. Cell Dev. Biol..

[34] Mijanović L., Putar D., Mimica L., Klajn S., Filić V., Weber I. (2025). The IQGAP-related RasGAP IqgC regulates cell–substratum adhesion in *Dictyostelium discoideum*. Cell. Mol. Biol. Lett..

[35] Ingham R.J., Colwill K., Howard C., Dettwiler S., Lim C.S.H., Yu J., Kadjia H., Raajimakers J., Gish G., Mbamalu G. (2005). WW domains provide a platform for the assembly of multiprotein networks. Mol. Cell. Biol..

[36] Iglesias-Bexiga M., Castillo F., Cobos E.S., Oka T., Sudol M., Luque I. (2015). WW domains of the YAP transcriptional regulator behave as independent units with different binding preferences for PPxY motif-containing ligands. PLoS ONE.

[37] Bardwell A.J., Lagunes L., Zebarjedi R., Bardwell L. (2017). The WW domain of the scaffolding protein IQGAP1 is neither necessary nor sufficient for binding to ERK1 and ERK2. J. Biol. Chem..

[38] Wang K., Okada H., Wloka C., Bi E. (2023). Unraveling the mechanisms and evolution of a two-domain module in IQGAP proteins for controlling eukaryotic cytokinesis. Cell Rep..

[39] Scheffzek K., Shivalingaiah G. (2019). Ras-specific GTPase-activating proteins—Structures, mechanisms, and interactions. Cold Spring Harb. Perspect. Med..

[40] Leblanc V., Tocque B., Delumeau I. (1998). Ras-GAP controls Rho-mediated cytoskeletal reorganization through its SH3 domain. Mol. Cell. Biol..

[41] Reimer M., Denby E., Zustiak S.P., Schober J.M. (2017). Ras GAP-related and C-terminal domain-dependent localization and tumorigenic activities of IQGAP1 in melanoma cells. PLoS ONE.

[42] Nouri K., Fansa E.K., Amin E., Dvorsky R., Gremer L., Willbold D., Schmitt L., Timson D.J., Ahmadian N.R., Schober J.M. (2016). IQGAP1 interaction with RHO family proteins revisited: Kinetic and equilibrium evidence for multiple distinct binding sites. J. Biol. Chem..

[43] Su Z., Dhusia K., Wu Y. (2020). Understanding the functions of scaffold proteins in cell signaling by a mesoscopic simulation method. Biophys. J..

[44] Rahmat M.B., Hussain A., Teh Y.X., Dutta B., Pundrik S., Kappei D., Ito Y. (2025). Wnt target IQGAP3 promotes Wnt signaling via disrupting Axin1–CK1α interaction. Oncogene.

[45] Goto T., Sato A., Adachi S., Iemura S.I., Natsume T., Shibuya H. (2013). IQGAP1 protein regulates nuclear localization of β-catenin via importin-β5 protein in Wnt signaling. J. Biol. Chem..

[46] White C.D., Erdemir H.H., Sacks D.B. (2012). IQGAP1 and its binding proteins control diverse biological functions. Cell. Signal..

[47] McNulty D.E., Li Z., White C.D., Sacks D.B., Annan R.S. (2011). MAPK scaffold IQGAP1 binds the EGF receptor and modulates its activation. J. Biol. Chem..

[48] Owen D., Campbell L.J., Littlefield K., Evatts K.A., Li Z., Sacks D.B., Lowe P.N., Mott H.R. (2008). The IQGAP1–Rac1 and IQGAP1–Cdc42 interactions. J. Biol. Chem..

[49] Monteleon C.L., McNeal A., Duperret E.K., Oh S.J., Schapira E., Ridky T.W. (2015). IQGAP1 and IQGAP3 serve individually essential roles in normal epidermal homeostasis and tumor progression. J. Investig. Dermatol..

[50] Mosaddeghzadeh N., Pudewell S., Bazgir F., Kazemein Jasemi N.S., Krumbach O.H.F., Gremer L., Willbold D., Dvorsky R., Ahmadian M.R. (2022). CDC42-IQGAP Interactions Scrutinized: New Insights into the Binding Properties of the GAP-Related Domain. Int. J. Mol. Sci..

[51] Rittmeyer E.N., Daniel S., Hsu S.C., Osman M.A. (2008). A dual role for IQGAP1 in regulating exocytosis. J. Cell Sci..

[52] Sakurai-Yageta M., Recchi C., Le Dez G., Sibarita J.B., Daviet L., Camonis J., D’Souza-Schroey C., Chavrier P. (2008). The interaction of IQGAP1 with the exocyst complex is required for tumor cell invasion downstream of Cdc42 and RhoA. J. Cell Biol..

[53] Kuroda S., Fukata M., Nakagawa M., Fujii K., Nakamura T., Ookubo T., Izawa I., Nagase T., Nomura N., Tani H. (1998). Role of IQGAP1, a target of the small GTPases Cdc42 and Rac1, in regulation of E-cadherin-mediated cell–cell adhesion. Science.

[54] Brandt D.T., Grosse R. (2007). Get to grips: Steering local actin dynamics with IQGAPs. EMBO Rep..

[55] Deng S., Cheng D., Wang J., Wang J., Gu J., Xue Y., Jiang Z., Qin L., Mao F., Cao Y. (2023). MYL9 expressed in cancer-associated fibroblasts regulates the immune microenvironment of colorectal cancer and promotes tumor progression in an autocrine manner. J. Exp. Clin. Cancer Res..

[56] Zhu S., Zou Y., Guo J., Ma W., Lu L., Liu R., Kang J., Zhao K., Zhong J. (2025). IQGAP1: Cross-disease target via receptor-pathway networks. Front. Oncol..

[57] Nouri K., Timson D.J., Ahmadian M.R. (2017). New model for the interaction of IQGAP1 with CDC42 and RAC1. Small GTPases.

[58] Cheung K.L., Lee J.H., Shu L., Kim J.H., Sacks D.B., Kong A.N.T. (2013). The Ras GTPase-activating-like protein IQGAP1 mediates Nrf2 protein activation via the MEK–ERK pathway. J. Biol. Chem..

[59] Roy M., Li Z., Sacks D.B. (2005). IQGAP1 is a scaffold for Mitogen-activated protein kinase signaling. Mol. Cell. Biol..

[60] Casar B., Crespo P. (2016). ERK signals: Scaffolding scaffolds?. Front. Cell Dev. Biol..

[61] Zhou G., Yang J., Song P. (2018). Correlation of ERK/MAPK signaling pathway with proliferation and apoptosis of colon cancer cells. Oncol. Lett..

[62] Gocke C.B., McMillan R., Wang Q., Begum A., Penchev V.R., Ali S.A., Borrello I., Huff C.A., Matsui W. (2016). IQGAP1 scaffold–MAP kinase interactions enhance multiple myeloma clonogenic growth and self-renewal. Mol. Cancer Ther..

[63] Liu Y., Yang Y., Wang X., Yin S., Liang B., Zhang Y., Fan M., Fu Z., Shen C., Han Y. (2024). Function of microRNA-124 in the pathogenesis of cancer (Review). Int. J. Oncol..

[64] Sanuki R., Yamamura T. (2021). Tumor suppressive effects of miR-124 and its function in neuronal development. Int. J. Mol. Sci..

[65] Sun Y., Zhao X., Luo M., Zhou Y., Ren W., Wu K., Li X., Shen J., Hu Y. (2014). The pro-apoptotic role of the regulatory feedback loop between miR-124 and PKM1/HNF4α in colorectal cancer cells. Int. J. Mol. Sci..

[66] Xi Z.W., Xin S.Y., Zhou L.Q., Yuan H.X., Wang Q., Chen K.X. (2015). Downregulation of rho-associated protein kinase 1 by miR-124 in colorectal cancer. World J. Gastroenterol..

[67] Fan J., Zhang W., Wu Y., Wan P., Guo Q., Zhang Y. (2018). MiR-124 inhibits cell growth through targeting IQGAP1 in colorectal cancer. Mol. Med. Rep..

[68] Hu W., Wang Z., Zhang S., Lu X., Wu J., Yu K., Ji A., Lu W., Wang Z., Wu J. (2019). IQGAP1 promotes pancreatic cancer progression and epithelial–mesenchymal transition through Wnt/β-catenin signaling. Sci. Rep..

[69] Jin X., Liu Y., Liu J., Lu W., Liang Z., Zhang D., Liu G., Zhu H., Xu N., Liang S. (2015). The overexpression of IQGAP1 and β-catenin is associated with tumor progression in hepatocellular carcinoma in vitro and in vivo. PLoS ONE.

[70] Zeng S., Wang J., Shi Z., Zhao H., Gao J., Li J. (2025). The Wnt/β-catenin signaling pathway in colorectal cancer: Mechanism and intervention of traditional Chinese medicine and chemical compounds. Front. Pharmacol..

[71] Zhang T., Wang Z., Liu Y., Huo Y., Liu H., Xu C., Mao R., Zhu Y., Liu L., Wei D. (2020). Plastin 1 drives metastasis of colorectal cancer through the IQGAP1/Rac1/ERK pathway. Cancer Sci..

[72] Yang C., Xiong Y., Wang D., Shi J. (2026). Identification of a novel PLS1 heterozygous variant causing autosomal dominant non-syndromic hearing loss. Exp. Ther. Med..

[73] David Y., Castro I.G., Yifrach E., Bibi C., Katawi E., Har-Shai D.Y., Bordsky S., Barkai N., Ravid T., Eisenstein M. (2022). Pls1 is a peroxisomal matrix protein with a role in regulating lysine biosynthesis. Cells.

[74] Rivero F., Santella L., Kozole S.L., Beningo K.A. (2024). Myosin light chains in the progression of cancer. Cells.

[75] Hoeprich G.J., Sinclair A.N., Shekhar S., Goode B.L. (2022). Single-molecule imaging of IQGAP1 regulating actin filament dynamics. Mol. Biol. Cell.

[76] Epp J.A., Chant J. (1997). An IQGAP-related protein controls actin-ring formation and cytokinesis in yeast. Curr. Biol..

[77] Deng C., Xie C., Li Z., Mei J., Wang K. (2025). Multi-omics analysis identifies diagnostic circulating biomarkers and potential therapeutic targets, revealing IQGAP1 as an oncogene in gastric cancer. npj Precis. Oncol..

[78] Tang T., Wang J., Zhang L., Cheng Y., Saleh L., Gu Y., Zhang H. (2021). IQGAP2 acts as an independent prognostic factor and is related to immunosuppression in DLBCL. BMC Cancer.

[79] Kumar D., Hassan M.K., Pattnaik N., Mohapatra N., Dixit M. (2017). Reduced expression of IQGAP2 and higher expression of IQGAP3 correlates with poor prognosis in cancers. PLoS ONE.

[80] Deng Z., Wang L., Hou H., Zhou J., Li X. (2016). Epigenetic regulation of IQGAP2 promotes ovarian cancer progression via activating Wnt/β-catenin signaling. Int. J. Oncol..

[81] Jin S.H., Akiyama Y., Fukamachi H., Yanagihara K., Akashi T., Yuasa Y. (2008). IQGAP2 inactivation through aberrant promoter methylation and promotion of invasion in gastric cancer cells. Int. J. Cancer.

[82] Zimak J., Wagoner Z.W., Nelson N., Waechtler B., Schlosser H., Kopecky N., Wu J., Zhao W. (2021). Epigenetic silencing directs expression heterogeneity of stably integrated multi-transcript unit genetic circuits. Sci. Rep..

[83] Gnatenko D.V., Xu X., Zhu W., Schmidt V.A. (2013). Transcript profiling identifies Iqgap2^−/−^ mouse as a model for advanced human hepatocellular carcinoma. PLoS ONE.

[84] Xu L., Shao Y., Ren L., Liu X., Li Y., Xu J., Ye Y. (2020). IQGAP2 inhibits migration and invasion of gastric cancer cells via elevating SHIP2 phosphatase activity. Int. J. Mol. Sci..

[85] He P.Y., Yip W.K., Chai B.L., Chai B.Y., Jabar M.F., Dusa N., Mohtarrudin N., Seow H.F. (2017). Inhibition of cell migration and invasion by miR-29a-3p in a colorectal cancer cell line through suppression of CDC42BPA mRNA expression. Oncol. Rep..

[86] Song F., Dai Q., Grimm M.O., Steinbach D. (2023). The antithetic roles of IQGAP2 and IQGAP3 in cancers. Cancers.

[87] Mo W.Y., Cao S.Q. (2022). MiR-29a-3p: A potential biomarker and therapeutic target in colorectal cancer. Clin. Transl. Oncol..

[88] Wang B., Li W., Yang L., Liao Q., Cui S., Wang H., Zhao L. (2014). miR-29b suppresses tumor growth and metastasis in colorectal cancer via downregulating Tiam1 expression and inhibiting epithelial-mesenchymal-transition. Cell Death Dis..

[89] Ghaleb A.M., Bialkowska A.B., Snider A.J., Gnatenko D.V., Hannum Y.A., Yang V.W., Schmidt V.A. (2015). IQ motif-containing GTPase-activating protein 2 (IQGAP2) is a novel regulator of colonic inflammation in mice. PLoS ONE.

[90] Liu X., Huang H., Remmers N., Hollingsworth M.A. (2014). Loss of E-cadherin and epithelial-to-mesenchymal transition is not required for cell motility in tissues or for metastasis. Tissue Barriers.

[91] Lialios P., Alimperti S. (2025). Role of E-cadherin in epithelial barrier dysfunction: Implications for bacterial infection, inflammation, and disease pathogenesis. Front. Cell. Infect. Microbiol..

[92] Petrova Y.I., Schecterson L., Gumbiner B.M. (2016). Roles for E-cadherin cell surface regulation in cancer. Mol. Biol. Cell.

[93] Dai Q., Song F., Li X., Huang F., Zhao H. (2022). Comprehensive analysis of the expression and prognosis for IQ motif-containing GTPase-activating proteins in hepatocellular carcinoma. BMC Cancer.

[94] Shimura M., Matsuo J., Pang S.C., Jangphattananont N., Hussain A., Rahat M.B., Lee J.W., Douchi D., Tong J.J.L., Myint K. (2025). IQGAP3 signalling mediates intratumoral functional heterogeneity to enhance malignant growth. Gut.

[95] Fang X., Zhang B., Thisse B., Bloom G.S., Thisse C. (2015). IQGAP3 is essential for cell proliferation and motility during zebrafish embryonic development. Cytoskeleton.

[96] Myint K., Chuang L.S.H., Teh Y.X., Mawan N.A., Shi E.J., Mok M.M.H., Nuttonmanit N., Matsuo J., Li Y., Yang H. (2022). Oncofetal protein IGF2BP1 regulates IQGAP3 expression to maintain stem cell potential in cancer. iScience.

[97] Xu W., Xu B., Yao Y., Yu X., Cao H., Zhang J., Liu J., Sheng H. (2016). Overexpression and biological function of IQGAP3 in human pancreatic cancer. Am. J. Transl. Res..

[98] Wu J., Chen Z., Cao H., Yu Z., Feng J., Wang K., Lu Q., Wu Y. (2019). High expression of IQGAP3 indicates poor prognosis in colorectal cancer patients. Int. J. Biol. Markers.

[99] Hu J., Neiswinger J., Zhang J., Zhu H., Qian J. (2015). Systematic prediction of scaffold proteins reveals new design principles in scaffold-mediated signal transduction. PLoS Comput. Biol..

[100] Shaw A.S., Filbert E.L. (2009). Scaffold proteins and immune-cell signalling. Nat. Rev. Immunol..

[101] Matsuo J., Douchi D., Myint K., Mon N.N., Yamamura A., Kohu K., Heng D.K.L., Chen S., Mawan N.A., Nuttonmanit N. (2021). Iqgap3–Ras axis drives stem cell proliferation in the stomach corpus during homeostasis and repair. Gut.

[102] Wei T., Choi S., Buehler D., Anderson R.A., Lambert P.F. (2019). A PI3K/AKT scaffolding protein, IQ motif-containing GTPase associating protein 1 (IQGAP1), promotes head and neck carcinogenesis. Clin. Cancer Res..

[103] Hervieu A., Heuss S.F., Zhang C., Barrow-McGee R., Joffre C., Ménard L., Clarke P.A., Kermorgant S. (2020). A PI3K- and GTPase-independent Rac1–mTOR mechanism mediates MET-driven anchorage-independent cell growth but not migration. Sci. Signal..

[104] Mammana S., Bramanti P., Mazzon E., Cavalli E., Basile M.S., Fagone P., Petralia M.C., McCubrey J.A., Nicoletti F., Mangano K. (2018). Preclinical evaluation of the PI3K/Akt/mTOR pathway in animal models of multiple sclerosis. Oncotarget.

[105] Glaviano A., Foo A.S.C., Lam H.Y., Yap K.C.H., Jacot W., Jones R.H., Eng H., Nair M.G., Makvandi P., Geoerger B. (2023). PI3K/AKT/mTOR signaling transduction pathway and targeted therapies in cancer. Mol. Cancer.

[106] Su J., Li H. (2015). RAC1 overexpression promotes the proliferation, migration and epithelial–mesenchymal transition of lens epithelial cells. Int. J. Clin. Exp. Pathol..

[107] Zhou H., Li X.M., Meinkoth J., Pittman R.N. (2000). Akt regulates cell survival and apoptosis at a postmitochondrial level. J. Cell Biol..

[108] Aseervatham J. (2020). Cytoskeletal remodeling in cancer. Biology.

[109] Yang Y., Zhao W., Xu Q.W., Wang X.S., Zhang Y., Zhang J. (2014). IQGAP3 promotes EGFR–ERK signaling and the growth and metastasis of lung cancer cells. PLoS ONE.

[110] Jinawath N., Shiao M.S., Chanpanitkitchote P., Svasti J., Furukawa Y., Nakamura Y. (2020). Enhancement of migration and invasion of gastric cancer cells by IQGAP3. Biomolecules.

[111] Small J.V., Stradal T., Vignal E., Rottner K. (2002). The lamellipodium: Where motility begins. Trends Cell Biol..

[112] Budi H.S., Anitasari S., Shen Y.K., Yamada S. (2024). Cytoskeletal regulation on polycaprolactone/graphene porous scaffolds for bone tissue engineering. Sci. Rep..

[113] Dianat-Moghadam H., Azizi M., Eslami-S Z., Cortés-Hernández L.E., Heidarifard M., Nouri M., Alix-Panabières C. (2020). The role of circulating tumor cells in the metastatic cascade: Biology, technical challenges, and clinical relevance. Cancers.

[114] Sanchez-Laorden B., Viros A., Marais R. (2013). Mind the IQGAP. Cancer Cell.

[115] Mohapatra T., Dixit M. (2022). IQ motif containing GTPase activating proteins (IQGAPs), A-kinase anchoring proteins (AKAPs) and kinase suppressor of Ras proteins (KSRs) in scaffolding oncogenic pathways and their therapeutic potential. ACS Omega.

[116] Hayashi H., Nabeshima K., Aoki M., Hamasaki M., Enatsu S., Yamauchi Y., Yamashita Y., Iwasaki H. (2010). Overexpression of IQGAP1 in advanced colorectal cancer correlates with poor prognosis: Critical role in tumor invasion. Int. J. Cancer.

[117] Su D., Liu Y., Song T. (2017). Knockdown of IQGAP1 inhibits proliferation and epithelial–mesenchymal transition by Wnt/β-catenin pathway in thyroid cancer. OncoTargets Ther..

[118] Mustafa M., Ahmad R., Tantry I.Q., Ahmad W., Siddiqui S., Alam M., Abbas K., Moinuddin, Hassan I., Habib S. (2024). Apoptosis: A comprehensive overview of signaling pathways, morphological changes, and physiological significance and therapeutic implications. Cells.

[119] Yakubov R., Kaloti R., Persaud P., McCracken A., Zadeh G., Bunda S. (2025). It’s all downstream from here: RTK/RAF/MEK/ERK pathway resistance mechanisms in glioblastoma. J. Neurooncol..

[120] Bahar M.E., Kim H.J., Kim D.R. (2023). Targeting the RAS/RAF/MAPK pathway for cancer therapy: From mechanism to clinical studies. Signal Transduct. Target. Ther..

[121] Zeke A., Lukács M., Lim W.A., Reményi A. (2009). Scaffolds: Interaction platforms for cellular signalling circuits. Trends Cell Biol..

[122] Qiao D., Wang R.C., Wang Z. (2025). Precision oncology: Current landscape, emerging trends, challenges, and future perspectives. Cells.

[123] Horikawa M., Hayase J., Kamakura S., Kohda A., Nakamura M., Sumimoto H. (2024). The scaffold protein IQGAP1 promotes reorientation of epithelial cell polarity at the two-cell stage for cystogenesis. Genes Cells.

